# Ensemble machine learning for predicting in-hospital mortality in Asian women with ST-elevation myocardial infarction (STEMI)

**DOI:** 10.1038/s41598-024-61151-x

**Published:** 2024-05-29

**Authors:** Sazzli Kasim, Putri Nur Fatin Amir Rudin, Sorayya Malek, Khairul Shafiq Ibrahim, Wan Azman Wan Ahmad, Alan Yean Yip Fong, Wan Yin Lin, Firdaus Aziz, Nurulain Ibrahim

**Affiliations:** 1 Cardiology Department, Faculty of Medicine, Universiti Teknologi MARA (UiTM), Shah Alam, Malaysia; 2 Cardiac Vascular and Lung Research Institute, Universiti Teknologi MARA (UiTM), Shah Alam, Malaysia; 3 National Heart Association of Malaysia, Heart House, Kuala Lumpur, Malaysia; 4 Institute of Biological Sciences, Faculty of Science, University Malaya, Kuala Lumpur, Malaysia; 5 Division of Cardiology, University Malaya Medical Centre (UMMC), Kuala Lumpur, Malaysia; 6 Department of Cardiology, Sarawak General Hospital, Kuching, Sarawak Malaysia; 7 Faculty of Medicine, Universiti Teknologi MARA (UiTM), Sungai Buloh Campus, Sungai Buloh, Malaysia; 8 School of Liberal Studies, Universiti Kebangsaan Malaysia, Bangi, Malaysia

**Keywords:** Computational biology and bioinformatics, Cardiology

## Abstract

The accurate prediction of in-hospital mortality in Asian women after ST-Elevation Myocardial Infarction (STEMI) remains a crucial issue in medical research. Existing models frequently neglect this demographic's particular attributes, resulting in poor treatment outcomes. This study aims to improve the prediction of in-hospital mortality in multi-ethnic Asian women with STEMI by employing both base and ensemble machine learning (ML) models. We centred on the development of demographic-specific models using data from the Malaysian National Cardiovascular Disease Database spanning 2006 to 2016. Through a careful iterative feature selection approach that included feature importance and sequential backward elimination, significant variables such as systolic blood pressure, Killip class, fasting blood glucose, beta-blockers, angiotensin-converting enzyme inhibitors (ACE), and oral hypoglycemic medications were identified. The findings of our study revealed that ML models with selected features outperformed the conventional Thrombolysis in Myocardial Infarction (TIMI) Risk score, with area under the curve (AUC) ranging from 0.60 to 0.93 versus TIMI's AUC of 0.81. Remarkably, our best-performing ensemble ML model was surpassed by the base ML model, support vector machine (SVM) Linear with SVM selected features (AUC: 0.93, CI: 0.89–0.98 versus AUC: 0.91, CI: 0.87–0.96). Furthermore, the women-specific model outperformed a non-gender-specific STEMI model (AUC: 0.92, CI: 0.87–0.97). Our findings demonstrate the value of women-specific ML models over standard approaches, emphasizing the importance of continued testing and validation to improve clinical care for women with STEMI.

## Introduction

Premenopausal women typically exhibit a lower risk of ST-elevation myocardial infarction (STEMI), but this risk escalates with age and the emergence of cardiovascular disease (CVD) risk factors, leading to more severe outcomes compared to men^1–3^. Studies indicate a higher in-hospital mortality rate for women with STEMI, as well as a higher prevalence of comorbidities such as hypertension, diabetes, and obesity. However, most randomized clinical trials have limited female representation, which raises concerns about the relevance of their findings^4–7^.

Risk scoring systems such as Thrombolysis in Myocardial Infarction (TIMI) and the Global Registry of Acute Coronary Events (GRACE) are vital for predicting STEMI mortality. However, they are largely based on the Western population from the 1990s and early 2000s, inadequately representing the diverse Asian population^8–10^. Furthermore, discrepancies in risk factors, such as differing smoking rates among Western and Asian females with acute myocardial infarction (AMI), and atypical AMI symptoms in women, limit their global applicability^11^. Additionally, their reliance on logistic regression (LR) presents limitations like rigid data assumptions. These issues underscore the need for new methods tailored to predict mortality in Asian female STEMI patients^12–14^.

Machine learning (ML), with its diverse statistical techniques and algorithms, presents a powerful alternative to traditional risk-scoring systems, enabling computers to learn from data and enhance decision-making and performance without explicit programming in healthcare ^15–18^. These include ML algorithms such as LR, support vector machine (SVM), k-nearest neighbours (KNN), decision tree (DT), random forest (RF), extreme gradient boosting (XGBoost), and adaptive boosting (AdaBoost)^19–22^. These algorithms have been especially beneficial for patient subgroups defined by specific characteristics such as age and comorbid diabetes, giving superior area under the curve (AUC) metrics than traditional methods^9,23–27^.

Despite ML's growing presence in cardiology, research focused on STEMI in Asian women remains limited. Studies have been reported on risk factors in multi-ethnic cohorts and age-related CVD patterns using ML algorithms; however, gender-specific ML-based models are scarce^28,29^. This gap highlights the urgent need for gender-specific ML models in cardiology tailored to Asian women with STEMI.

Ensemble ML, an advanced ML method, combines multiple models to improve predictive accuracy and adaptability, which is very useful in healthcare's complex environment^16^. Its application is evident in CVD studies, where studies using ensemble ML show better illness prediction accuracy and patient outcomes^30–32^. Ensemble ML is reported to outperform single ML algorithms, which is crucial in medical fields where precision impacts patient survival^33^. Feature selection techniques further optimize ML models in healthcare, essential for identifying mortality risk factors in high-risk STEMI patients^34,35^. However limited studies have been reported on ensemble ML and feature selection methods of women in STEMI.

Addressing the underrepresentation of Asian women in STEMI-related ML models, our study explores both base and ensemble ML models, employing six established algorithms like SVM, KNN, DT, RF, XGBoost, and AdaBoost as base learners. We also focus on identifying key factors associated with in-hospital mortality among multi-ethnic Asian women, a demographic often neglected in existing models, using ML feature selection methods. We aim to compare traditional risk scores with both base ML and advanced ensemble ML models, employing feature selection techniques rooted in RF and SVM algorithms. This also involves analysing our models against diverse registry data and evaluating a model specifically tailored for women against a more general model encompassing all STEMI patients. Ultimately, our goal is to improve prediction accuracy, fostering more personalized and effective clinical decision-making for Asian women with STEMI.

## Materials and methods

### Study design and setting

We conducted a retrospective cohort analysis using anonymised data from the National Cardiovascular Disease Database (NCVD-ACS) from 2006 to 2016. The NCVD, which is supported by the Ministry of Health Malaysia (MOH) and the National Heart Association of Malaysia (NHAM), collects detailed information on patients diagnosed with Acute Coronary Syndrome (ACS), which includes conditions like STEMI and non-ST segment elevation myocardial infarction (NSTEMI). It includes a wide range of patient information from 24 collaborating Malaysian hospitals, including demographics, treatments, and medications ^36^.

**Figure 1:**
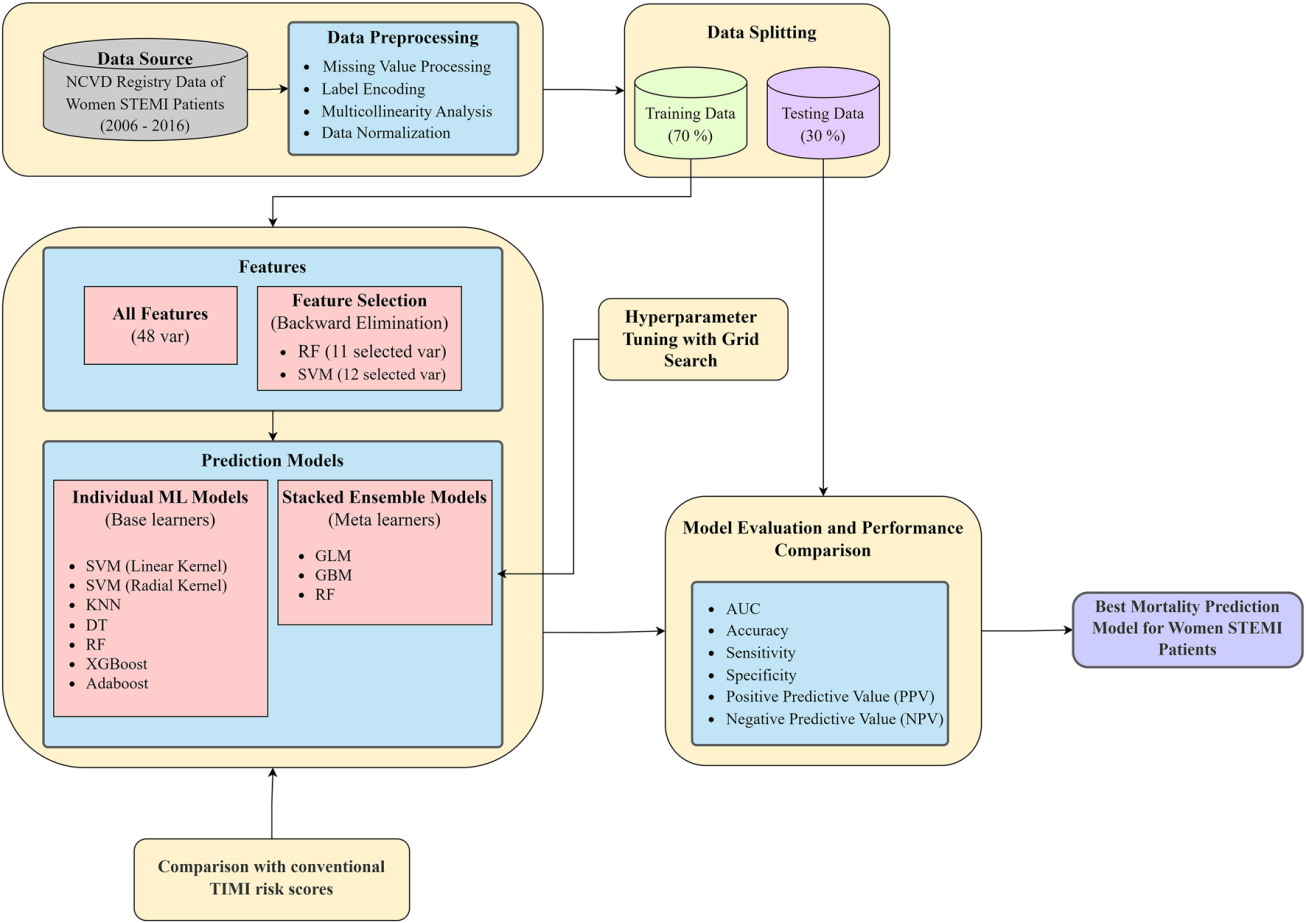
Research workflow and methodology applied in this study.

The study focused on female STEMI patients, to address a research gap in this demographic, particularly in Malaysia. The data gathered from a network of healthcare facilities in both urban and rural areas, represents an extensive and robust sample for research. The study proposes the application of advanced ML techniques to construct predictive models tailored to the unique epidemiological profiles of Asian women with STEMI, hence improving the personalization and effectiveness of their clinical care. The study's workflow and methods are shown in Fig. 1.

### Participants

The cohort for this study was collected from the NCVD-ACS registry and spanned the years 2006 to 2016. Our primary analysis included primarily female STEMI patients’ complete data records for clinical outcome analysis. For our secondary analysis, we increased the scope by incorporating three distinct datasets to enhance the robustness and generalizability of our findings:

- Women complete dataset: consisting of female patients with complete data, allowing a focused analysis on the intended demographic with no missing values in predictor variables.
- Women imputed dataset: including a larger dataset with missing values addressed through multivariable imputation, increasing female patient records to represent a broader range of clinical circumstances.
- General complete dataset: including complete data for both male and female STEMI patients, which provides a comparative perspective across genders and allows us to examine the model's performance in a broader context.

### Data Source

Our study utilized anonymized patient data from the NCVD-ACS registry spanning from 2006 to 2016. Consecutive in-hospital STEMI cases comprised a total of 15,407 with 6299 complete cases identified (with no missing values on predictors). This study utilised 871 cases of female patients for primary analysis using complete cases from a total of 6299 datasets.

In 2007, the Medical Review & Ethics Committee (MREC) of the MOH of Malaysia approved the NCVD registry study (Approval Code: NMRR-07-20-250). The MREC waived patient informed consent for NCVD^37,38^. This study also has been authorized by the UiTM ethics committee (Reference number: 600-TNCPI (5/1/6)) and NHAM. The data used in this study were made anonymous before use, as in our research data are interested only in the values and features without having access to patient personal information.

The dataset used in this study includes each patient's information at the time of STEMI hospitalization. Based on the data available at the time, predictions for in-hospital mortality were developed, with the model being utilized once per patient. During the hospital stay, no more predictions were made, aligning the prediction frequency with the crucial decision-making period at the time of patient admission.

### Variables and data preprocessing

#### Variables

STEMI was defined as persistent ST-segment elevation ≥ 1 mm in two contiguous electrocardiographic leads, or the presence of a new left bundle branch block in the setting of positive cardiac markers^39^. Input variables are features that are used as input in the development of a model to predict the outcome (in-hospital mortality). 48 variables (9 continuous, 39 categorical) from a complete set of data were used in this study (Supplementary Table 1). The categories of variables used were sociodemographic characteristics, CVD diagnosis and severity, CVD risk factors, CVD comorbidities, non-CVD comorbidities, clinical presentation, baseline investigation, electrocardiography (ECG), treatments, and pharmacological therapy. Variables used for model development are variables in the emergency department as first contact as well as variables in the hospital. Our study adopts the following method to address the dynamic nature of patient data during hospitalization:

- Clinical history, examination, and investigation findings: based on information obtained at the time of admission, these provide a baseline understanding of each patient's initial status.
- Treatment: we include the initial medical responses and interventions, as well as the primary treatment administered during hospitalization.
- Medication: recognizing that medication regimens can change, our models consider the final pharmaceutical regimen recommended before discharge, capturing any substantial changes in treatment.
- Outcome variable (in-hospital mortality): determined based on the patient's condition at the time of discharge, providing a specific endpoint for each case.

The mortality period begins on the day of hospital admission. For in-hospital mortality, the calculation period began with the first hospital admission. Through record links with the Malaysian National Registration Department, the death was confirmed. The registry does not collect information on short-term complications, such as heart failure. Planned follow-up data points were intended to collect this information, but we omitted them from this study due to the high rate of missing values. To increase the significance of this study, we centred our algorithm on policy-altering endpoints such as death. This was accomplished in similar publications^9,40,41^. The missing rates for each variable utilised in this study are presented in Supplementary Table 1.

#### Data splitting

We used the stratified random sampling to separate the dataset for model development (70%) and validation (30%) based on Kuhn and Johnson study^42^ to avoid data leakage^43^. In circumstances of multiple admissions, a unique patient identification ensured that each patient's data was consistently labelled as the training or testing set, preserving anonymity^44^.

Data pre-processing methods such as imputation (on missing cases) and balancing (both complete and missing cases) were performed on training data only. Meanwhile, normalization methods were done separately on both training and testing data. We accessed the performance of the developed model and TIMI using a validation set that accounts for 30% of data that is not used for model development.

#### Data balancing

Our dataset had a significant class imbalance, with non-survival cases (n = 73) accounting for approximately 8.38% of the total dataset (n = 871) and survival cases (n = 798) accounting for 91.62%. To mitigate the imbalance issue and improve the robustness of our model, we used the ROSE package to combine up-sampling and down-sampling techniques on the training data^45^. The class distribution was adjusted to better reflect a balanced scenario, improving the reliability of subsequent analyses and the predictive performance of the developed models. To preserve the integrity and representativeness of real-world clinical scenarios, this treatment was not applied to the validation dataset.

#### Data imputation

Since our dataset is prospective, the proportion of missing values across all variables was arbitrary and out of our hands. The definition of an incomplete dataset is up to 30% of variables missing. The probability of missing data in our dataset is independent of both observed values and unseen data components. Our dataset is classified as missing completely at random, indicating that the distribution of missing values is random and independent of any variable that may or may not be included in the analysis. We performed multivariable imputation using chained equations and predicted mean matching from the MICE R package to deal with missing cases for the secondary analysis ^46^. This method imputes missing values using actual values from other cases in which predicted values are the closest.

#### Data normalization

Data normalization was used to reduce the bias of features that contribute more numerically to pattern class discrimination^42^. We employed standardization or z-score normalization, for continuous variables (age, heart rate, systolic and diastolic blood pressure, total cholesterol, high-density lipoproteins (HDL), low-density lipoproteins (LDL), triglyceride, fasting blood glucose) in this study.

### Data analysis

#### Primary analysis

A total of 6299 in-hospital STEMI complete cases were identified (with no missing values on predictors). 871 cases of woman patients were extracted from the data and used as the final dataset for primary analysis. This rendered a full predictor set of 48 variables (9 continuous, 39 categorical) for the study as shown in Table 1.

**Table 1:** Summary statistics of the complete and imputed dataset.

Variables (n = 48)	Description	Complete dataset				Imputed dataset (training data)			
		All Cases (n = 871)	Survival (798)	Non-survival (73)	p-value	All Cases (n = 2003)	Survival (n = 1727)	Non-survival (n = 256)	p-value
Age		62.2 ± 11.4	61.8 ± 11.5	67 ± 9.8	**<0.001**	63.7 ± 11.6	63 ± 11.6	68.5 ± 11	0
Race	1: Malay	465 (53.39)	416 (52.13)	49 (67.12)	0.020	1072 (53.52)	921 (52.72)	151 (58.98)	0.005
	2: Chinese	162 (18.6)	149 (18.67)	13 (17.81)		390 (19.47)	337 (19.29)	53 (20.7)	
	3: Indian	205 (23.54)	199 (24.94)	6 (8.22)		411 (20.52)	369 (21.12)	42 (16.41)	
	4: Others	39 (4.48)	34 (4.26)	5 (6.85)		130 (6.49)	120 (6.87)	10 (3.91)	
Smoking status	1: Never	787 (90.36)	717 (89.85)	70 (95.89)	0.005	1762 (87.97)	1528 (87.46)	234 (91.41)	0.022
	2: Former	33 (3.79)	31 (3.88)	2 (2.74)		100 (4.99)	89 (5.09)	11 (4.3)	
	3: Current	51 (5.86)	50 (6.27)	1 (1.37)		141 (7.04)	130 (7.44)	11 (4.3)	
History of hypertension	1: Yes	632 (72.56)	573 (71.8)	59 (80.82)	0.069	1479 (73.84)	1280 (73.27)	199 (77.73)	0.113
	2: No	239 (27.44)	225 (28.2)	14 (19.18)		524 (26.16)	467 (26.73)	57 (22.27)	
History of diabetes	1: Yes	499 (57.29)	447 (56.02)	52 (71.23)	0.008	1191 (59.46)	1033 (59.13)	158 (61.72)	0.428
	2: No	372 (42.71)	351 (43.98)	21 (28.77)		812 (40.54)	714 (40.87)	98 (38.28)	
History of myocardial infarction	1: Yes	64 (7.35)	51 (6.39)	13 (17.81)	0.015	223 (11.13)	190 (10.88)	33 (12.89)	0.366
	2: No	807 (92.65)	747 (93.61)	60 (82.19)		1780 (88.87)	1557 (89.12)	223 (87.11)	
History of chronic angina	1: Yes	62 (7.12)	52 (6.52)	10 (13.7)	0.087	184 (9.19)	159 (9.1)	25 (9.77)	0.738
	2: No	809 (92.88)	746 (93.48)	63 (86.3)		1819 (90.81)	1588 (90.9)	231 (90.23)	
History of heart failure	1: Yes	25 (2.87)	20 (2.51)	5 (6.85)	0.156	99 (4.94)	70 (4.01)	29 (11.33)	**<0.001**
	2: No	846 (97.13)	778 (97.49)	68 (93.15)		1904 (95.06)	1677 (95.99)	227 (88.67)	
History of chronic lung disease	1: Yes	23 (2.64)	17 (2.13)	6 (8.22)	0.067	53 (2.65)	43 (2.46)	10 (3.91)	0.256
	2: No	848 (97.36)	781 (97.87)	67 (91.78)		1950 (97.35)	1704 (97.54)	246 (96.09)	
History of renal disease	1: Yes	47 (5.4)	35 (4.39)	12 (16.44)	0.008	140 (6.99)	107 (6.12)	33 (12.89)	0.002
	2: No	824 (94.6)	763 (95.61)	61 (83.56)		1863 (93.01)	1640 (93.88)	223 (87.11)	
History of cerebrovascular disease	1: Yes	37 (4.25)	31 (3.88)	6 (8.22)	0.194	103 (5.14)	88 (5.04)	15 (5.86)	0.599
	2: No	834 (95.75)	767 (96.12)	67 (91.78)		1900 (94.86)	1659 (94.96)	241 (94.14)	
Family history of premature CVD	1: Yes	88 (10.1)	86 (10.78)	2 (2.74)	**<0.001**	195 (9.74)	179 (10.25)	16 (6.25)	0.018
	2: No	783 (89.9)	712 (89.22)	71 (97.26)		1808 (90.26)	1568 (89.75)	240 (93.75)	
Heart rate (bpm)		85.3 ± 21.7	84.7 ± 20.9	92.2 ± 28.5	0.031	86.2 ± 22.2	85.6 ± 21.4	90.3 ± 26.6	0.008
**Systolic blood pressure (mmHg)**		136.8 ± 28.5	138.2 ± 28.2	121.5 ± 26.7	**<0.001**	135.4 ± 31.8	137.4 ± 31.1	121.5 ± 33.5	**<0.001**
Diastolic blood pressure (mmHg)		79 ± 17.7	79.6 ± 17.6	72.6 ± 18.2	0.002	77.8 ± 19.8	78.6 ± 19.6	72 ± 20.4	**<0.001**
**Killip classification**	1: Killip I	561 (64.41)	539 (67.54)	22 (30.14)	**<0.001**	1140 (56.91)	1073 (61.42)	67 (26.17)	**<0.001**
	2: Killip II	158 (18.14)	146 (18.3)	12 (16.44)		379 (18.92)	320 (18.32)	59 (23.05)	
	3: Killip III	48 (5.51)	42 (5.26)	6 (8.22)		150 (7.49)	127 (7.27)	23 (8.98)	
	4: Killip IV	104 (11.94)	71 (8.9)	33 (45.21)		334 (16.67)	227 (12.99)	107 (41.8)	
Total cholesterol (mmol/L)		5.4 ± 1.8	5.5 ± 1.8	5.2 ± 1.8	0.180	5.5 ± 11.8	5.6 ± 12.7	4.8 ± 1.6	0.010
HDL (mmol/L)		1.2 ± 1	1.2 ± 1	1.1 ± 0.4	0.006	1.4 ± 5.6	1.4 ± 6	1.1 ± 0.5	0.098
LDL (mmol/L)		3.5 ± 1.7	3.5 ± 1.7	3.3 ± 1.6	0.324	3.7 ± 10.6	3.7 ± 11.3	3.3 ± 1.5	0.098
Triglyceride (mmol/L)		1.7 ± 1.1	1.7 ± 1.1	1.8 ± 1	0.792	2.2 ± 12.6	2.1 ± 12.7	2.8 ± 11.9	0.337
**Fasting blood glucose (mmol/L)**		9.7 ± 4.9	9.4 ± 4.7	12.6 ± 6.5	**<0.001**	10 ± 5.9	9.8 ± 5.9	11.3 ± 5.5	**<0.001**
ST-segment elevation ≥ 1mm in ≥ 2 contiguous limb leads	1: Selected	405 (46.5)	363 (45.49)	42 (57.53)	0.051	990 (49.43)	871 (49.86)	119 (46.48)	0.314
	2: Not selected	466 (53.5)	435 (54.51)	31 (42.47)		1013 (50.57)	876 (50.14)	137 (53.52)	
ST-segment elevation ≥ 2mm in ≥ 2 contiguous frontal leads	1: Selected	498 (57.18)	461 (57.77)	37 (50.68)	0.252	1081 (53.97)	936 (53.58)	145 (56.64)	0.358
	2: Not selected	373 (42.82)	337 (42.23)	36 (49.32)		922 (46.03)	811 (46.42)	111 (43.36)	
ST-segment depression ≥ 0.5mm in ≥ 2 contiguous leads	1: Selected	106 (12.17)	93 (11.65)	13 (17.81)	0.189	234 (11.68)	203 (11.62)	31 (12.11)	0.823
	2: Not selected	765 (87.83)	705 (88.35)	60 (82.19)		1769 (88.32)	1544 (88.38)	225 (87.89)	
T-wave inversion ≥ 1mm	1: Selected	60 (6.89)	54 (6.77)	6 (8.22)	0.666	147 (7.34)	132 (7.56)	15 (5.86)	0.290
	2: Not selected	811 (93.11)	744 (93.23)	67 (91.78)		1856 (92.66)	1615 (92.44)	241 (94.14)	
Abnormal bundle branch block (BBB)	1: Selected	22 (2.53)	19 (2.38)	3 (4.11)	0.474	67 (3.34)	51 (2.92)	16 (6.25)	0.035
	2: Not selected	849 (97.47)	779 (97.62)	70 (95.89)		1936 (96.66)	1696 (97.08)	240 (93.75)	
Inferior leads: II, III, aVF	1: Selected	435 (49.94)	401 (50.25)	34 (46.58)	0.551	984 (49.13)	869 (49.74)	115 (44.92)	0.150
	2: Not selected	436 (50.06)	397 (49.75)	39 (53.42)		1019 (50.87)	878 (50.26)	141 (55.08)	
Anterior leads: V1 to V4	1: Selected	442 (50.75)	405 (50.75)	37 (50.68)	0.991	1026 (51.22)	878 (50.26)	148 (57.81)	0.023
	2: Not selected	429 (49.25)	393 (49.25)	36 (49.32)		977 (48.78)	869 (49.74)	108 (42.19)	
Lateral leads: 1, aVL, V5 to V6	1: Selected	201 (23.08)	175 (21.93)	26 (35.62)	0.021	441 (22.02)	370 (21.18)	71 (27.73)	0.028
	2: Not selected	670 (76.92)	623 (78.07)	47 (64.38)		1562 (77.98)	1377 (78.82)	185 (72.27)	
True posterior: V1, V2	1: Selected	83 (9.53)	73 (9.15)	10 (13.7)	0.279	173 (8.64)	150 (8.59)	23 (8.98)	0.835
	2: Not selected	788 (90.47)	725 (90.85)	63 (86.3)		1830 (91.36)	1597 (91.41)	233 (91.02)	
Right ventricle: ST elevation in lead V4R	1: Selected	73 (8.38)	64 (8.02)	9 (12.33)	0.284	149 (7.44)	125 (7.16)	24 (9.38)	0.250
	2: Not selected	798 (91.62)	734 (91.98)	64 (87.67)		1854 (92.56)	1622 (92.84)	232 (90.62)	
Cardiac catheterization	1: Yes	357 (40.99)	333 (41.73)	24 (32.88)	0.131	690 (34.45)	622 (35.6)	68 (26.56)	0.003
	2: No	514 (59.01)	465 (58.27)	49 (67.12)		1313 (65.55)	1125 (64.4)	188 (73.44)	
Percutaneous coronary intervention (PCI)	1: Yes	281 (32.26)	264 (33.08)	17 (23.29)	0.066	563 (28.11)	513 (29.36)	50 (19.53)	**<0.001**
	2: No	590 (67.74)	534 (66.92)	56 (76.71)		1440 (71.89)	1234 (70.64)	206 (80.47)	
Fibrinolytic status	1: Yes	601 (69)	547 (68.55)	54 (73.97)	0.321	1351 (67.45)	1174 (67.2)	177 (69.14)	0.53234
	2: No	270 (31)	251 (31.45)	19 (26.03)		652 (32.55)	573 (32.8)	79 (30.86)	
Aspirin	1: Yes	858 (98.51)	788 (98.75)	70 (95.89)	0.232	1918 (95.76)	1695 (97.02)	223 (87.11)	**<0.001**
	2: No	13 (1.49)	10 (1.25)	3 (4.11)		85 (4.24)	52 (2.98)	33 (12.89)	
Glycoprotein receptor inhibitor	1: Yes	20 (2.3)	20 (2.51)	0.00	**<0.001**	65 (3.25)	58 (3.32)	7 (2.73)	0.59734
	2: No	851 (97.7)	778 (97.49)	73 (100)		1938 (96.75)	1689 (96.68)	249 (97.27)	
Unfractionated heparin	1: Yes	138 (15.84)	125 (15.66)	13 (17.81)	0.649	316 (15.78)	276 (15.8)	40 (15.62)	0.943
	2: No	733 (84.16)	673 (84.34)	60 (82.19)		1687 (84.22)	1471 (84.2)	216 (84.38)	
Low Molecular Weight Heparin (LMWH)	1: Yes	235 (26.98)	217 (27.19)	18 (24.66)	0.635	625 (31.2)	551 (31.54)	74 (28.91)	0.388
	2: No	636 (73.02)	581 (72.81)	55 (75.34)		1378 (68.8)	1196 (68.46)	182 (71.09)	
**Beta-blocker**	1: Yes	544 (62.46)	524 (65.66)	20 (27.4)	**<0.001**	1167 (58.26)	1091 (62.45)	76 (29.69)	**<0.001**
	2: No	327 (37.54)	274 (34.34)	53 (72.6)		836 (41.74)	656 (37.55)	180 (70.31)	
**ACE inhibitor**	1: Yes	444 (50.98)	427 (53.51)	17 (23.29)	**<0.001**	966 (48.23)	904 (51.75)	62 (24.22)	**<0.001**
	2: No	427 (49.02)	371 (46.49)	56 (76.71)		1037 (51.77)	843 (48.25)	194 (75.78)	
Angiotensin II receptor blocker	1: Yes	33 (3.79)	33 (4.14)	0 (0.00)	**<0.001**	92 (4.59)	86 (4.92)	6 (2.34)	0.017
	2: No	838 (96.21)	765 (95.86)	73 (100.00)		1911 (95.41)	1661 (95.08)	250 (97.66)	
Statin	1: Yes	827 (94.95)	765 (95.86)	62 (84.93)	0.013	1800 (89.87)	1600 (91.59)	200 (78.12)	**<0.001**
	2: No	44 (5.05)	33 (4.14)	11 (15.07)		203 (10.13)	147 (8.41)	56 (21.88)	
Other lipid lowering agents	1: Yes	20 (2.3)	19 (2.38)	1 (1.37)	0.494	56 (2.8)	51 (2.92)	5 (1.95)	0.313
	2: No	851 (97.7)	779 (97.62)	72 (98.63)		1947 (97.2)	1696 (97.08)	251 (98.05)	
Diuretics	1: Yes	247 (28.36)	220 (27.57)	27 (36.99)	0.115	592 (29.56)	510 (29.19)	82 (32.03)	0.363
	2: No	624 (71.64)	578 (72.43)	46 (63.01)		1411 (70.44)	1237 (70.81)	174 (67.97)	
Calcium antagonist	1: Yes	63 (7.23)	59 (7.39)	4 (5.48)	0.502	171 (8.54)	151 (8.64)	20 (7.81)	0.647
	2: No	808 (92.77)	739 (92.61)	69 (94.52)		1832 (91.46)	1596 (91.36)	236 (92.19)	
**Oral hypoglycaemic agent**	1: Yes	240 (27.55)	231 (28.95)	9 (12.33)	**<0.001**	512 (25.56)	479 (27.42)	33 (12.89)	**<0.001**
	2: No	631 (72.45)	567 (71.05)	64 (87.67)		1491 (74.44)	1268 (72.58)	223 (87.11)	
Insulin	1: Yes	367 (42.14)	334 (41.85)	33 (45.21)	0.585	855 (42.69)	750 (42.93)	105 (41.02)	0.562
	2: No	504 (57.86)	464 (58.15)	40 (54.79)		1148 (57.31)	997 (57.07)	151 (58.98)	
Anti-arrhythmic agent	1: Yes	42 (4.82)	38 (4.76)	4 (5.48)	0.797	162 (8.09)	131 (7.5)	31 (12.11)	0.032
	2: No	829 (95.18)	760 (95.24)	69 (94.52)		1841 (91.91)	1616 (92.5)	225 (87.89)	

The asterisk (*) with p-value < 0.001 indicated that the variable difference between the alive and dead group is statistically significant.

Significant values are given in bold.

#### Secondary analysis

Secondary analyses on the best-performing algorithm were carried out;

(i) For the 15,407 STEMI cases with missing data, we employed multivariable imputation using chained equations to estimate missing values, creating a comprehensive dataset for modelling. This allowed us to include a total of 2197 additional female patients in our analysis, broadening the scope and applicability of our results.

(ii) A total of 4369 patients out of 6299 in-hospital STEMI patients with complete cases, including both male and female patients, were used to train the algorithm with the best performance. Both a women-specific model and a population-specific model were tested and compared using identical testing datasets (262 cases) from the primary analysis of all cases.

#### Additional statistics

This study presents the mean and standard deviation (SD) of continuous variables as well as the frequencies of categorical variables. Correlation analysis revealed variable associations. Univariate analysis used a Chi-Square test to find significant variables and a two-sided independent student t-test (p < 0.05) to compare them. Pair-wise corrected resampled t-tests were used to compare the base and ensemble ML model performance ^49,67^. A p-value less than 0.001 indicated statistical significance.

### Feature selection

RF and SVM algorithms have produced better results than other base learners in this study. Hence, ranked features from RF and SVM algorithms were used for feature selection. The sequential backward elimination (SBE) algorithm removes irrelevant features in ascending order using model significance value^47^. Iteratively, SBE was applied to RF and SVM-ranked variables in ascending order^48^. The prediction models were trained and evaluated for each iteration using the 30% validation dataset that was not used for model development. The models' predictive performance was calculated, and the models with the highest performance and fewest variables were chosen. Then, the base and ensemble ML models were constructed using the selected features from RF and SVM.

### Model development

#### Base ML algorithms

ML algorithms such as SVM^52^, KNN^53^, DT^54^, RF^54^, XGBoost^55^, and AdaBoost^56^ were used to develop prediction models for women with STEMI in R (Version 4.1.2).

SVM is a robust learning algorithm that was used in this study in conjunction with both a linear and a radial basis function (RBF) kernel. KNN is a simple supervised machine learning algorithm that has seen widespread use in the healthcare industry for classification and regression problems (Bansal et al., 2018). DT is a non-parametric supervised learning technique used for classification and regression. To generate multiple small decision trees, RF employs bagging with DT as the primary classifier. The models use the class with the most votes predicted by RF trees. XGB is an implementation of gradient boosting. Gradient Boosting with XGB is more regularised, which improves model generalisation and prevents overfitting, resulting in a more precise result. AdaBoost is an adaptive learning algorithm because it transforms weak learners into strong learners through multiple iterations. These algorithms were chosen based on previous CVD mortality-related research^22,24,27,28,57,58^. All the hyper-parameters utilised in the development of base and ensemble ML models were tuned using a combination of random search and manual tuning (refer to Supplementary Table 2).

#### Ensemble ML algorithms

Stacking, a type of ensemble ML algorithm, is a meta-learning strategy that uses the predictions of multiple base learners as input for training a new meta-learner, which makes the final prediction. It is more effective than any individual algorithm in classification and regression problems. In this study, six commonly used ML algorithms, including SVM, KNN, DT, RF, XGBoost, and AdaBoost, are used as base learners, followed by three commonly used meta learners, including RF, generalised logistic model (GLM), and generalized boosted models (GBM)^59–61^. 10-fold cross-validation was used to avoid overfitting for model development on the training set^49^.

### Model evaluation

Model calibration was evaluated using standardized measures on untouched raw validation dataset^62^. The primary evaluation metric, the AUC, was chosen based on research establishing its effectiveness in a wide range of class distributions, including imbalanced datasets ^63,64^. While AUC-PR provides more granularity for minority class predictive performance, AUC is still a widely accepted measure for overall diagnostic accuracy. Additional metrics included accuracy, sensitivity, specificity, positive predictive value (PPV), and negative predictive value (NPV), which provide a comprehensive view of model performance across both classes. To compare the predictive performance of ML models, a paired resampled t-test was used^65^. In addition, the net reclassification index (NRI) was calculated to determine the percentage improvement in identifying both positive and negative cases with the best model compared to the TIMI risk score^66^.

### Results interpretation

Due to their black-box nature, it is difficult to implement ML models in clinical medicine. Since ML models are agnostic, perturbing input and observing predictions can reveal the behaviour of the underlying model^50^. Modifying components that are understandable by humans enables us to interpret the input. Thus, we interpret the best ML model in this study using local interpretable model-agnostic explanations (LIME)^51^. LIME employs a simple linear model to approximate a black-box model locally, as opposed to globally.

### Comparative analysis

The computed TIMI scores obtained from the NCVD registry were utilized for validating the performance of the data. Using the 30% validation set data, the TIMI score was compared to the developed base and ensemble ML models using AUC. A performance breakdown graph was also created to evaluate the performance of the TIMI score based on clinical practice and literature cut-off points.

Validation of data was NCVD registry calculated TIMI scores were used for validation data performance. Using a validation set that was not used for model development, the AUC of TIMI score performance was compared to the developed base and ensemble ML models. A graph was also created to compare performance with the TIMI score based on clinical practice and literature cut-off points. The ML high-risk population for this study is defined by a mortality probability of greater than 50%, which is equivalent to a TIMI score of greater than 5.

### Ethical declaration

This study was authorized by the UiTM Research Ethics Committee (Reference: 600-TNCPI (5/1/6)), with the approval code REC/673/19. The UiTM Ethics Committee conducts following the ICH Good Clinical Practice Guidelines, Malaysia Good Clinical Practice Guidelines and Declaration of Helsinki.

## Results

### Patient characteristics

The characteristics of patients utilised in this study are detailed in Table 1. In the complete cases dataset, the mean age of in-hospital female STEMI survivors is 61.8 (SD 11.5) years, while the mean age of non-survivors is 67 (SD 9.8) years. Nearly 90% of the patients were non-smokers. 73% of the patients have a hypertension history, and 57% have diabetes. 32% of patients received percutaneous coronary intervention (PCI) treatment. The reported overall hospital mortality rate for women was 8.4%.

Table 1 also displays the summary statistics for the imputed dataset. The overall mortality rate for women was 12.8 %. There were significant differences in systolic blood pressure, Killip class, fasting blood glucose, beta-blocker, ACE inhibitor, and oral hypoglycemic agent between survivors and non-survivors in both complete cases and imputed datasets (p < 0.001 for all).

### Feature selection

SBE feature selection methods were combined with ML algorithms SVM and RF to construct predictive models with optimal performance (refer to methods). The comparison between features selected by ML feature selection with TIMI risk score is illustrated in Table 2. Killip class, fasting blood glucose, age and systolic blood pressure, beta blocker and percutaneous coronary intervention were observed as common predictors in both ML feature selection models in this study. The best SVM Linear model was built using twelve features selected using SVM algorithm feature selection methods. Age, Killip class, and systolic blood pressure are common characteristics shared by the TIMI risk score for STEMI and the best model. The ranking of the selected features by variable importance is presented in Supplementary Table 3.

**Table 2:** Comparison between features selected by ML feature selection with TIMI risk score.

Variables	ML algorithm		TIMI
	Random forest (11 variables)	Support vector machines (12 variables)	
Abnormal BBB in ECG			×
Age	×	×	×
ACE inhibitors		×	
Aspirin	×		
Beta-blocker	×	×	
Diabetes mellitus			×
Fasting blood glucose	×	×	
Fibrinolytic status			
HDL			
Heart rate	×		×
History of Renal Disease			
Killip class	×	×	×
ECG abnormal at lateral leads	×		
Oral hypoglycemic agent		×	
Percutaneous coronary intervention	×	×	
Race		×	
Right Ventricle: ST elevation in lead V4R		×	
Systolic blood pressure	×	×	×
Triglyceride	×		
Time to treatment (< 4 h)			×
Weight			×

### Algorithm performance on complete cases

On the 30% validation dataset, the models constructed using complete sets (48 variables) and a reduced set of variables compared to the TIMI risk score demonstrated the highest predictive performance (Table 3). Except for base DT and ensemble GBM, most ML models outperformed TIMI risk scores for the prediction of STEMI in women. The model with the best performance was base SVM (SVM selected var; p < 0.001). Table 4 provides a detailed performance evaluation of ML models relative to the TIMI risk score.

**Table 3:** The AUC of TIMI risk score and ML models with and without feature selection based on a 30% validation dataset.

Models	The area under the ROC Curve (95% CI)		
	All features	SVM selected features	RF selected features
**Base SVM (linear kernel)**	0.89 (0.80–0.98)	**0.93 (0.89–0.98)**	0.90 (0.85–0.96)
Base SVM (RBF kernel)	0.87 (0.81–0.94)	0.90 (0.85–0.95)	0.83 (0.76–0.90)
Base DT	0.73 (0.62–0.83)	0.70 (0.59–0.81)	0.60 (0.44–0.76)
Base KNN	0.84 (0.76–0.92)	0.78 (0.67–0.88)	0.84 (0.73–0.95)
Base RF	0.86 (0.79–0.93)	0.85 (0.78–0.91)	0.88 (0.81–0.96)
Base XGBoost	0.86 (0.78–0.94)	0.82 (0.74–0.89)	0.83 (0.74–0.93)
Base Adaboost	0.85 (0.77–0.92)	0.76 (0.68–0.85)	0.86 (0.79–0.94)
Ensemble ML (GBM meta-learner)	0.77 (0.67–0.88)	0.76 (0.66–0.86)	0.74 (0.63–0.85)
Ensemble ML (GLM meta-learner)	0.88 (0.81–0.95)	0.87 (0.81–0.92)	0.86 (0.76–0.95)
Ensemble ML (RF meta-learner)	0.91 (0.87–0.96)	0.82 (0.75–0.90)	0.85 (0.77–0.93)
TIMI	0.80 (0.71–0.89)		

Significant values are in bold.

**Table 4:** Detailed performance metrics of ML models with and without feature selection for women STEMI patients.

Models	Accuracy (95% CI)	Sensitivity	Specificity	PPV	NPV	Mcnemar’s test (p-value)
All features (48)						
Base SVM (linear kernel)	0.83 (0.78–0.88)	0.86	0.83	0.32	0.99	<0.001
Base SVM (RBF kernel)	0.91 (0.86–0.94)	0.14	0.98	0.33	0.93	0.016
Base DT	0.73 (0.67–0.78)	0.64	0.73	0.18	0.96	<0.001
Base KNN	0.70 (0.64–0.75)	0.82	0.68	0.19	0.98	<0.001
Base RF	0.90 (0.85–0.93)	0.32	0.95	0.37	0.94	0.700
Base XGBoost	0.81 (0.75–0.85)	0.77	0.81	0.27	0.98	<0.001
Base AdaBoost	0.91 (0.87–0.94)	0.32	0.96	0.44	0.94	0.307
Ensemble ML (GBM meta-learner)	0.92 (0.88–0.95)	0.18	0.99	0.57	0.93	0.002
Ensemble ML (GLM meta-learner)	0.90 (0.86–0.93)	0.18	0.97	0.33	0.93	0.078
Ensemble ML (RF meta-learner)	0.92 (0.88–0.95)	0.27	0.98	0.55	0.94	0.029
SVM selected features (12)						
Base SVM (Linear Kernel)	0.77 (0.71–0.82)	0.96	0.75	0.26	0.99	<0.001
Base SVM (RBF Kernel)	0.856 (0.81–0.90)	0.68	0.87	0.33	0.97	<0.001
Base DT	0.67 (0.61–0.73)	0.64	0.68	0.15	0.95	<0.001
Base KNN	0.6 (0.63–0.74)	0.82	0.68	0.19	0.98	<0.001
Base RF	0.87 (0.82–0.91)	0.36	0.92	0.29	0.94	0.391
Base XGBoost	0.86 (0.81–0.90)	0.36	0.90	0.25	0.94	0.144
Base AdaBoost	0.86 (0.82–0.90)	0.27	0.92	0.23	0.93	0.617
Ensemble ML (GBM meta-learner)	0.89 (0.84–0.92)	0.18	0.95	0.25	0.93	0.361
Ensemble ML (GLM meta-learner)	0.88 (0.84–0.92)	0.27	0.94	0.29	0.93	1.000
Ensemble ML (RF meta-learner)	0.89 (0.85–0.93)	0.27	0.95	0.32	0.93	0.719
RF selected features (11)						
Base SVM (linear kernel)	0.79 (0.74–0.84)	0.91	0.78	0.27	0.99	<0.001
Base SVM (RBF Kernel)	0.88 (0.84–0.92)	0.27	0.94	0.29	0.93	1.000
Base DT	0.76 (0.70–0.81)	0.59	0.77	0.19	0.95	<0.001
Base KNN	0.67 (0.61–0.73)	0.86	0.65	0.19	0.98	<0.001
Base RF	0.91 (0.86–0.94)	0.55	0.94	0.44	0.96	0.424
Base XGBoost	0.86 (0.81–0.90)	0.68	0.87	0.33	0.97	<0.001
Base AdaBoost	0.90 (0.85–0.93)	0.32	0.95	0.37	0.94	0.700
Ensemble ML (GBM meta-learner)	0.91 (0.86–0.94)	0.23	0.97	0.39	0.93	0.110
Ensemble ML (GLM meta-learner)	0.91 (0.87–0.94)	0.41	0.96	0.47	0.95	0.677
Ensemble ML (RF meta-learner)	0.92 (0.88–0.95)	0.41	0.96	0.50	0.95	0.522

The predictive performance of ML models constructed with SVM-selected features (AUC ranging from 0.70 to 0.93) was better compared to that of models constructed with RF-selected features (AUC ranging from 0.60 to 0.90). There was a significant difference between the base SVM-Linear (SVM selected var) algorithm and the base SVM-Linear (RF selected var) algorithm (p < 0.001). Models constructed with the ensemble RF model (AUC: 0.91, CI: 0.87–0.96) perform the best among ensemble ML models (Table 2). However, the base SVM with the linear kernel (SVM selected var) algorithm demonstrated the highest predictive performance with a reduced number of predictors (12 predictors) for in-hospital prediction of STEMI patients (AUC = 0.93, 95% CI = 0.89 to 0.97) compared to other base and ensemble ML models.

### Secondary analysis on best performing model

The best performing ML models, base SVM (SVM selected var), were also trained on an imputed dataset and a general dataset (data with complete cases that are not gender-specific). Then, both types of models were evaluated utilizing the complete cases validation dataset. This enables a valid comparison between models constructed with imputed, general, and complete cases models (Table 5).

**Table 5:** Detailed performance metrics of best SVM Linear model (SVM selected var) on the imputed dataset and general dataset for STEMI women patients.

Model	AUC (95% CI)	Accuracy (95% CI)	Sensitivity	Specificity	PPV	NPV
**SVM linear (women complete dataset)**	**0.93 (0.89–0.98)**	**0.77 (0.71–0.82)**	**0.96**	**0.75**	**0.26**	**0.994**
SVM linear (women imputed dataset)	0.89 (0.81–0.97)	0.85 (0.80–0.89)	0.82	0.85	0.34	0.981
SVM linear (general complete dataset)	0.92 (0.87–0.97)	0.78 (0.73–0.83)	0.91	0.77	0.27	0.989

Significant values are in bold.

SVM (SVM selected var), trained on imputed datasets performed comparably to models trained on the complete dataset using a similar validation dataset of complete cases: SVM (SVM selected var) (AUC = 0.89, CI: 0.81–0.96 vs AUC = 0.93, CI: 0.89–0.98) (p = 0.540). There is no statistically significant difference between the SVM model (SVM selected var) using complete cases with the imputed model.

Using the complete cases validation dataset, the model trained with women's complete cases performed better compared to the models trained with complete cases data that are not gender specific: SVM (SVM selected var) (AUC = 0.93, CI: 0.89–0.98 vs AUC = 0.92, CI: 0.87–0.97) (p < 0.001).

### Model interpretation

**Figure 2:**
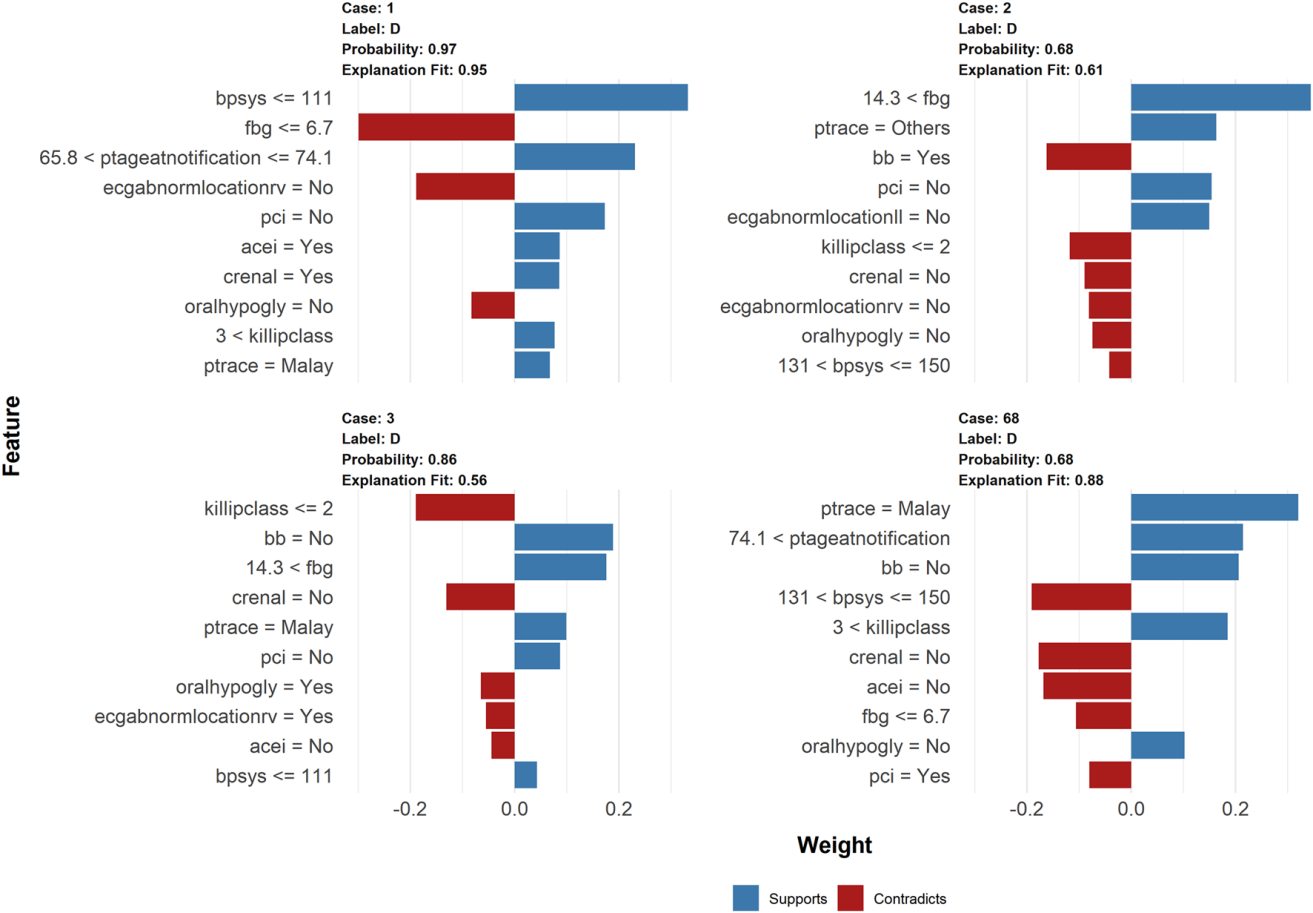
LIME model plots explaining individual predictions for dead cases.

**Figure 3:**
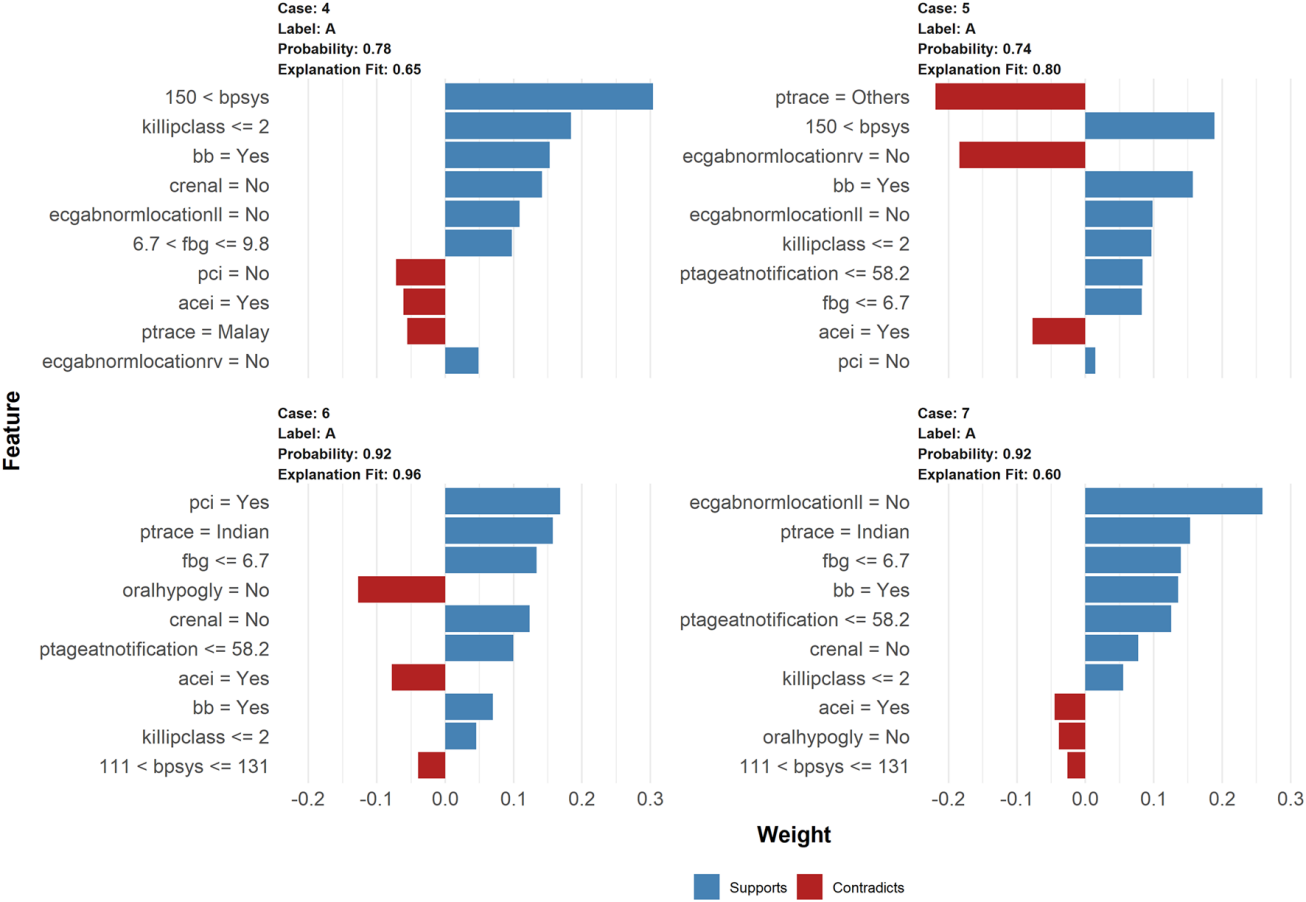
LIME model plots explaining individual predictions for alive cases.

LIME provides explanations for any individual patient, and the contribution of a given variable may change depending on other features of the patient. The contributions of the variables used for prediction by LIME analysis are illustrated for dead (Fig. 2) and alive (Fig. 3) cases respectively using the best performing model, base SVM Linear (SVM selected var) model.

Each graph illustrates the ten variables that best characterise the prediction in the local region. The blue bars represent variables that increase the predicted probability (supports), while the red bars represent variables that decrease the predicted probability (reduces) (contradicts). For instance, for the dead cases, a high Killip class > 3 and no PCI intervention with high systolic blood pressure (patient #1) or an older age > 74 years old (patient #68) are variables that strongly indicate non-survival. In the meantime, did not receive PCI intervention with high fasting blood sugar > 14.3 (patient #2) and older age > 74 with higher blood pressure (patient #3) were also strong indicators of non-survival. Pharmacological interventions are noted as variables that contradict and lower the predicted probability of non-survival in (patients #3 and #2). For patients who are alive (Fig. 3), a younger age of 58 years, the absence of chronic renal disease, a lower Killip class < 2, and a lower fasting blood glucose < 6.7 are all supportive of the survival outcome.

### Comparison with TIMI conventional risk score

**Figure 4:**
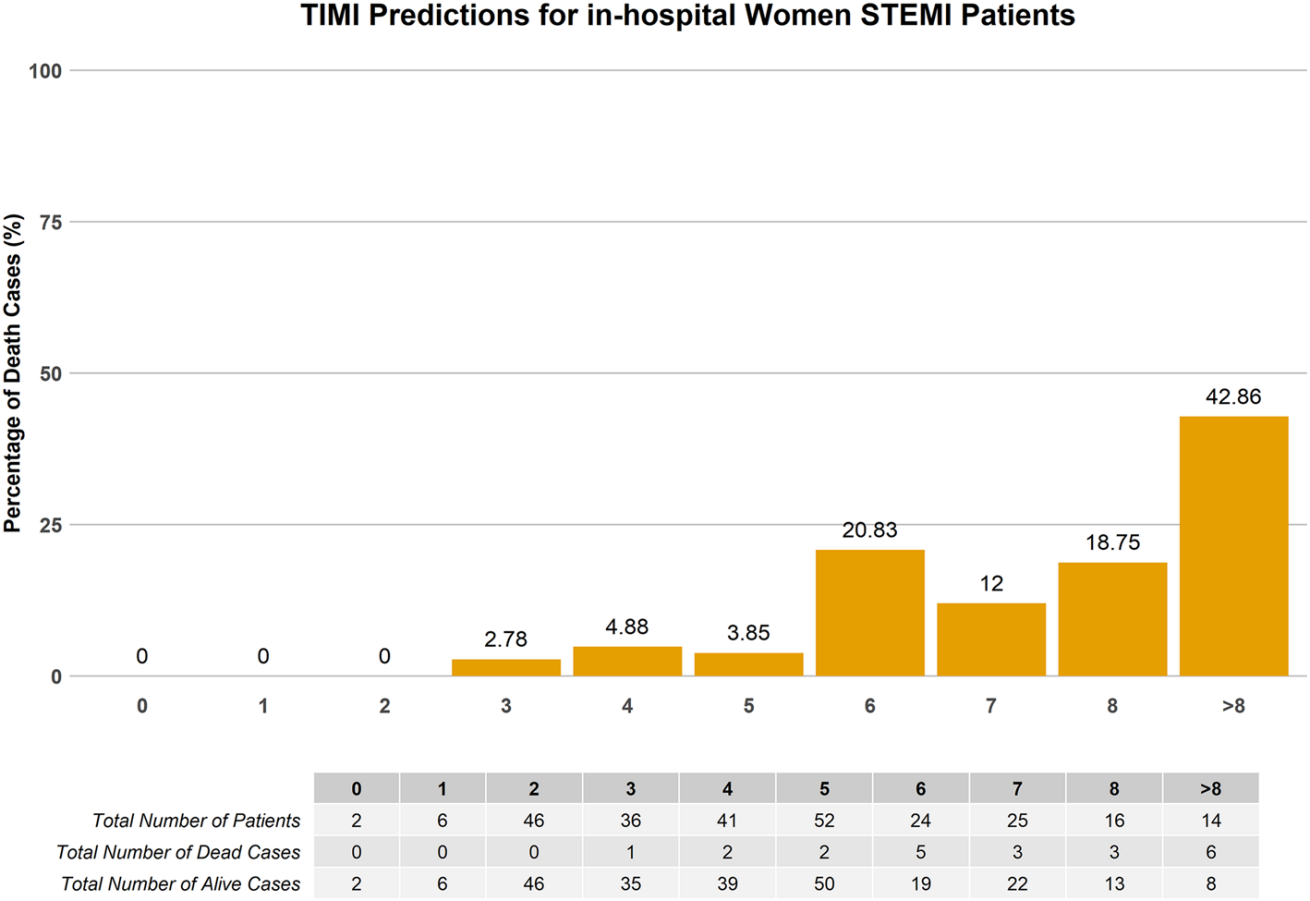
Mortality rate distribution on the validation set of TIMI risk scores.

**Figure 5:**
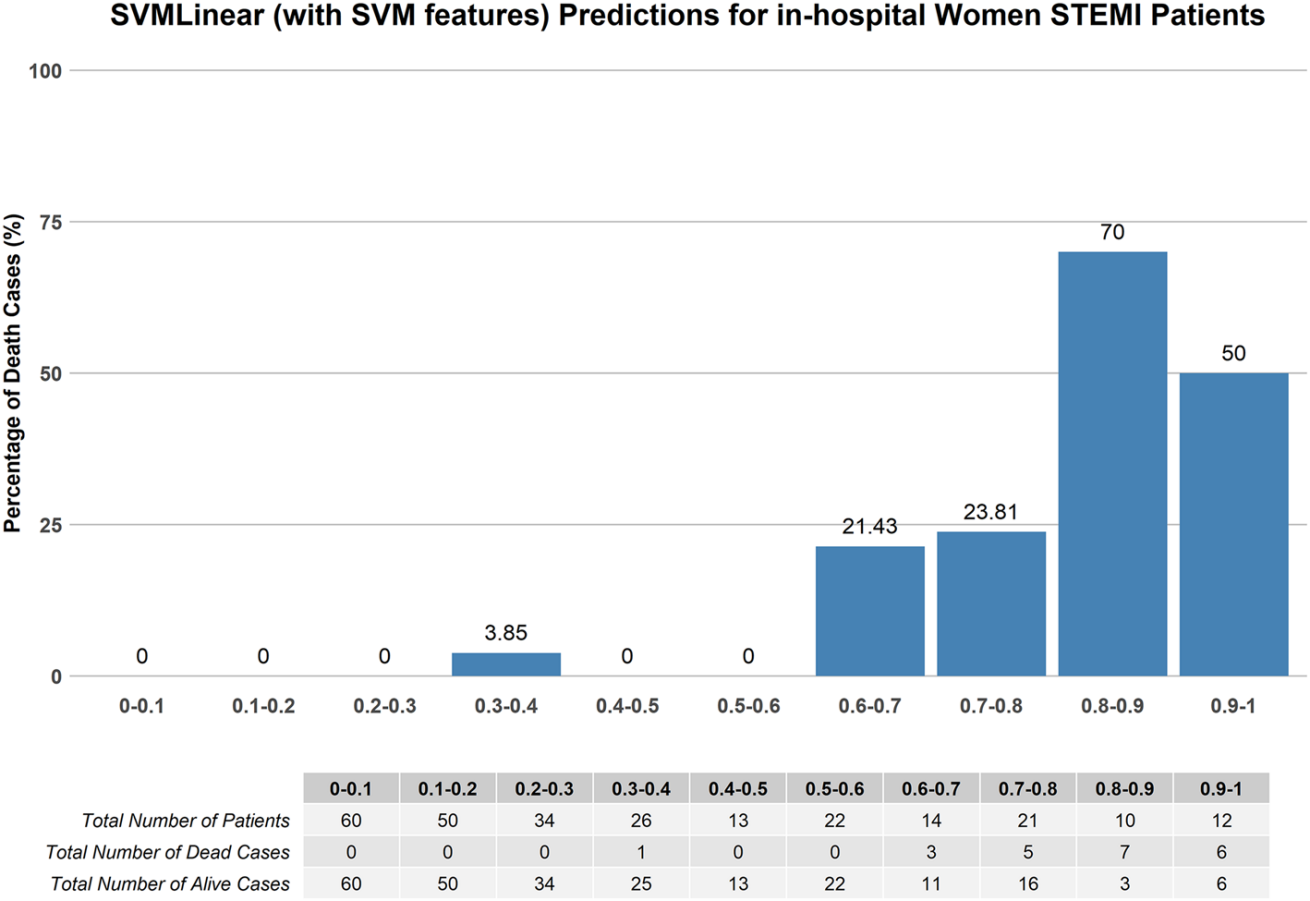
Mortality rate distribution on the validation set of base SVM (using SVM variables) model.

Using a similar validation set, TIMI achieved a lower AUC of 0.81 (0.72–0.89) compared to most of the ML models except for the base DT and ensemble GBM model. Figures 4 and 5 illustrate the graph plotted from the TIMI risk score and the best-performing model, base SVM Linear (SVM selected var) in predicting the mortality risk of the women STEMI patients respectively. For the women patients, the ML score categorized patients as low risk with the probability of < 50% and high-risk stratum as ≥ 50%. This is equivalent to a TIMI low-risk of score ≤ 5 and a high-risk score of > 5^68^.

Table 6 tabulates the percentage of mortality in the patients with predicted low risk (TIMI score: ≤ 5; ML probabilities < 0.5) and high risk (TIMI score: > 5; ML probabilities: ≥ 0.5). In the high-risk group, ML models predicted mortality better in comparison to TIMI for in-hospital death in women STEMI patients.

**Table 6:** Percentage of mortality in patients with predicted low risk (TIMI score: ≤ 5; ML probabilities < 0.5) and high risk (TIMI score: > 5; ML probabilities: ≥ 0.5).

Model	Low risk (%)	High risk (%)
TIMI risk scores	2.73	21.52
Base SVM linear (SVM selected variables)	0.55	25.93

### NRI analysis

NRI for the in-hospital model, the net reclassification of women STEMI patients using the base SVM (SVM selected var) produced a net reclassification improvement of 18.8% with p < 0.00001 over the original TIMI risk score.

**Table:** 

		Number of individuals		Reclassification		Net correctly reclassified (%)
		Machine learning		Increased risk	Decreased risk	
		Low risk	High risk			
	TIMI score					
Individuals with events (died) (n = 22)						
	Low risk	0	5	5	1	18
	High risk	1	16			
Individuals without events (alive) (n = 240)						
	Low risk	143	35	35	37	0.83
	High risk	37	25			
Net reclassification index (NRI)	18 + 0.83 = 18.83					
Z, p-value	Z= $\frac{18.83}{\sqrt{\frac{5+1 }{{22}^{2}}+\frac{35+37}{{240}^{2}}}}$ = 161.19 225.13, p < 0.00001					
Conclusion	It was statistically significant. ML model has a better predictive ability compared with the TIMI risk scores model in predicting the mortality rate of Asian women with STEMI patients, and the proportion of correct classification increased by **18.8%**					

## Discussion

This study developed and evaluated ML models to predict in-hospital mortality in Asian women with STEMI, comparing them with traditional risk scores like TIMI. Notably, it is the first study to apply ensemble ML models in this context, achieving higher accuracy than conventional risk scores. Key findings include: the crucial role of feature selection in enhancing model performance; identifying consistent predictors like systolic blood pressure, Killip class, fasting blood glucose, beta-blockers, ACE inhibitors, and oral hypoglycaemics medications; improved performance of ML models using selected features, the SVM linear model with SVM selected features showing the highest accuracy outperforming ensemble ML; most ML models, except DT and GBM, outperform TIMI score; and the use of LIME for model interpretability. These results underscore the value of advanced ML in specific clinical settings, enhancing predictive accuracy and decision-making in treating STEMI in Asian women.

Feature selection enhances ML model performance in our study, aligning with findings from Perez et al.^69^. Applications of feature selection algorithms increase ML model performance ^70–75^, as seen in this study with the RF (11 predictors) and SVM (12 predictors) models. However, this approach contrasts with other mortality post-STEMI studies where models using larger sets of predictors showed optimal performance ^35,76^. ML with significant predictors improves risk stratification in Asian STEMI women, providing clinicians with a prognostic tool for better emergency care management.

This study's findings also reveal that ensemble ML methods show promise in predicting in-hospital mortality for Asian female patients, though their performance did not consistently exceed that of base ML algorithms. Particularly, base learners like SVM (AUC: 0.93) and RF (AUC: 0.90) performed on par with ensemble ML models. In medical contexts, even small increases in predictive model performance are crucial^77^. However, it is notable that the ensemble ML method does not always outperform the base model^78^. This has been demonstrated in this study that the improvement of the ensemble ML model was not significantly greater than the best-performing base learners SVM, as demonstrated in the literature^27,50^.

The best-performed model, base SVM Linear managed to identify high-risk patients that reported higher mortality than those classified as high-risk in TIMI. Despite its widespread use in Asia, the TIMI risk score, originally developed from a predominantly Western Caucasian cohort, had limited Asian representation, and only included 25% female participants, indicating an underrepresentation of women. In our study, ML models validated against TIMI showed an AUC value of 0.81 in a non-restricted PCI eligible population, higher than the 0.78 AUC for the fibrinolytic eligible STEMI population reported in the original TIMI study^79^. The SVM algorithm's robustness in managing high-dimensional and constrained datasets renders it ideal for predicting in-hospital mortality, and its proficiency in modelling non-linear decision boundaries is beneficial for assessing severe AMI prognosis^80,81^.

NRI was further used for a detailed assessment of model enhancements compared to the TIMI score. The NRI, though less commonly reported in medical research, effectively measures how accurately a new model reclassifies individuals into appropriate risk categories^82^. In our study we achieved a significant 18.8% improvement in classification accuracy over the TIMI score, indicating that our ML models not only predict more precisely but also better reflect actual patient outcomes. Accuracy tests for NRI were conducted on a separate dataset from that used for model development, providing an unbiased comparison with TIMI and reinforcing the validity of our results.

Our ML models, using feature selection, identified age, Killip class, and systolic blood pressure as key predictors, aligning with univariate analysis and LIME. LIME analysis indicated that factors like older age, increased fasting blood glucose, and absence of percutaneous coronary intervention (PCI) were associated with higher mortality risk, consistent with existing research. However, LIME's identification of influential features should be seen as preliminary and not indicative of causality, necessitating further validation through prospective or randomized controlled trials^83,84^.

Older female STEMI patients have a higher incidence of coronary artery disease than males^2^, with Killip class being a key predictor of STEMI patients^6,85,86^. This finding is consistent with our study and previous ML-based mortality studies^40^. Women with STEMI face higher mortality due to factors like atypical symptoms, delayed treatment, and less frequent use of cardiac catheterization. Our study found only 34% of Asian STEMI patients received PCI, highlighting a need for improved care. Heart rate is a crucial factor in in-hospital mortality^87^, and the use of beta-blockers post-STEMI is linked to better outcomes^5,7,86,88^.

Several limitations exist in this study. Firstly, we could only validate ML models using only the TIMI score. Parameters to calculate the GRACE score were not acquired during patient admission compared to the TIMI score. The TIMI score is adopted during admission due to its simplicity and its development for short-term risk stratification, along with findings that its performance is similar to the GRACE score for predicting in-hospital mortality. Hence collecting information for two risk scores is redundant^89^.

Future research will aim to utilize high-performance computing and larger datasets for better predictive performance of ensemble techniques. ML models, reliant on data representativeness rather than medical expertise, may exhibit biases and require ongoing validation with real-world data, which can be facilitated by electronic health record systems in hospitals. Integrating these models into hospital systems for physician use and validating them in clinical registries rather than administrative databases, will be key areas of future investigation.

## Conclusion

This work demonstrates the effectiveness of both base and ensemble ML models, when combined with feature selection, in predicting in-hospital mortality in Asian women with STEMI. Our findings highlight the potential for combining these advanced ML models with conventional risk-scoring approaches like TIMI to improve mortality risk assessments in this specific group. This opens up the possibility of more nuanced and effective therapeutic decision-making. The improved predictive accuracy achieved by these models not only allows for better patient communication and awareness but also allows healthcare practitioners to optimize their management methods and resource allocation more effectively. In the future, incorporating these ML technologies into clinical practice could greatly enhance care for female STEMI patients. Furthermore, our findings pave the way for future research to test and potentially integrate these models into clinical processes, ultimately leading to more tailored and improved healthcare outcomes for women with STEMI.

### Supplementary Information

Supplementary Table 1.

Supplementary Table 2.

Supplementary Table 3.

## Data Availability

Data which support the findings of this research are accessible from the National Heart Association of Malaysia (NHAM), but the availability of these data is restricted, therefore they are not publicly available. It belongs to the individual ministry of health universities hospitals and private hospitals that require multiple institutional agreements for data release to third parties therefore ethical approval is required for analysis. Data are however available from NHAM upon request using https://www.malaysianheart.org/?p=contact or email them at secretariat@malaysianheart.org. Any findings from the data need to be reported and permission needs to be obtained from the NHAM committee before publication.
