# Supplementary material for: Ensemble machine learning for predicting in-hospital mortality in Asian women with ST-elevation myocardial infarction (STEMI)
Source: Sci Rep. 2024 May 29;14:12378. doi: 10.1038/s41598-024-61151-x (PMC11137033; doi:10.1038/s41598-024-61151-x)
Supplement: Supplementary file 1 — Supplementary Table 1. [file 41598_2024_61151_MOESM1_ESM.docx]

**Supplementary Table 1 : Variables Missing Rate**

| **Variables** | **Number of Missing Data** | **Missing Rate Percentage (%)** |
| --- | --- | --- |
| **Demographic** | | |
| Age | 0 | 0.00 |
| Race | 0 | 0.00 |
| **Status Before Event** | | |
| Smoking status | 588 | 3.83 |
| History of Hypertension | 2036 | 13.25 |
| History of Diabetes | 2219 | 14.44 |
| History of Myocardial Infarction | 2091 | 13.61 |
| History of Chronic Angina | 2464 | 16.04 |
| History of Heart Failure | 1847 | 12.02 |
| History of Chronic Lung Disease | 1876 | 12.21 |
| History of Renal Disease | 1851 | 12.05 |
| History of Cerebrovascular Disease | 1822 | 11.86 |
| Family History of Premature Cardiovascular Disease | 3130 | 20.37 |
| **Clinical Presentation & Examination** | | |
| Heart Rate (bpm) | 554 | 3.61 |
| Systolic Blood Pressure (mmHg) | 455 | 2.96 |
| Diastolic Blood Pressure (mmHg) | 562 | 3.66 |
| Killip Classifications | 677 | 4.41 |
| **Baseline Investigation (values obtained within 48 hours from admission)** | | |
| Total Cholesterol (mmol/L) | 3221 | 20.96 |
| HDL (mmol/L) | 3594 | 23.39 |
| LDL (mmol/L) | 3622 | 23.57 |
| Triglyceride (mmol/L) | 3654 | 23.78 |
| Fasting Blood Glucose (mmol/L) | 3416 | 22.23 |
| **Electrocardiography (ECG)** | | |
| Abnormalities type | | |
| ST-segment Elevation ≥1mm in ≥ 2 Contiguous Limb Leads | 0 | 0.00 |
| ST-segment Elevation ≥ 2mm in ≥ 2 Contiguous Frontal Leads | 0 | 0.00 |
| ST-segment Depression ≥ 0.5mm in ≥ 2 Contiguous Leads | 0 | 0.00 |
| T-wave inversion ≥1mm | 0 | 0.00 |
| Bundle Branch Block (BBB) | 0 | 0.00 |
| Abnormality location | | |
| Inferior Leads: II, III, aVF | 0 | 0.00 |
| Anterior Leads: V1 to V4 | 0 | 0.00 |
| Lateral Leads: 1, aVL, V5 to V6 | 0 | 0.00 |
| True Posterior: V1, V2 | 0 | 0.00 |
| Right Ventricle: ST Elevation in Lead V4R | 0 | 0.00 |
| **Invasive Therapeutic Procedures** | | |
| Cardiac Catherization | 604 | 3.93 |
| Percutaneous Coronary Intervention | 1261 | 8.21 |
| Fibrinolytic Status | 253 | 1.65 |
| **Pharmacological Therapy** | | |
| Aspirin | 337 | 2.19 |
| GP Receptor Inhibitor | 2320 | 15.10 |
| Unfractioned Heparin | 2187 | 14.23 |
| LMWH | 2066 | 13.45 |
| Beta-blocker | 1603 | 10.43 |
| ACE Inhibitor | 1687 | 10.98 |
| Angiotensin II Receptor Blocker | 2335 | 15.20 |
| Statin | 489 | 3.18 |
| Other lipid lowering agent | 2283 | 14.86 |
| Diuretics | 2011 | 13.09 |
| Calcium antagonist | 2317 | 15.08 |
| Oral Hyperglycemia | 2160 | 14.06 |
| Insulin | 1952 | 12.70 |
| Anti-arrhythmic Agent | 2343 | 15.25 |
| **Outcomes** | | |
| Patient Outcome | 0 | 0.00 |
