# Supplementary material for: Ensemble machine learning for predicting in-hospital mortality in Asian women with ST-elevation myocardial infarction (STEMI)
Source: Sci Rep. 2024 May 29;14:12378. doi: 10.1038/s41598-024-61151-x (PMC11137033; doi:10.1038/s41598-024-61151-x)
Supplement: Supplementary file 2 — Supplementary Table 2. [file 41598_2024_61151_MOESM2_ESM.docx]

**Supplementary Table 2 : Hyperparameters used in ML Model Development**

| **Models** | **Parameters** | | |
| --- | --- | --- | --- |
|  | **All Features** | **SVM Features** | **RF Features** |
| **SVM (Linear Kernel)** | Kernal : Linear  C : 1 | Kernal : Linear  C : 0.1426378 | Kernal : Linear  C : 0.1426378 |
| **SVM (Radial Kernel)** | Kernal : Radial  C : 8  Sigma : 0.01290921 | Kernal : Radial  C : 4.28673  Sigma : 0.1745246 | Kernal : Radial  C : 4.28673  Sigma : 0.3478335 |
| **Random Forest** | ntree : 1000  mtry : 2 | ntree : 1000  mtry : 6 | ntree : 1000  mtry : 2 |
| **Decision Tree** | cp : 0.02777778 | cp : 0.02777778 | cp : 0.01736111 |
| **KNN** | k : 23 | k : 21 | k : 21 |
| **Adaboost** | nIter : 500 | nIter : 368 | nIter : 434 |
| **XGBoost** | nrounds : 50  max_depth : 8  eta : 0.1  gamma : 0.1  colsample_bytree : 0.5  min_child_weight : 0.5  subsample : 0.8 | nrounds : 204  max_depth : 3  eta : 0.471859  gamma : 1.539564  colsample_bytree : 0.4074143  min_child_weight : 3  subsample : 0.7822796 | nrounds : 432  max_depth : 5  eta : 0.1630174  gamma : 2.769955  colsample_bytree : 0.5462395  min_child_weight : 2  subsample : 0.8258106 |
| **Stacked Ensemble (GLM)** | No hyperparameter can be tuned | | |
| **Stacked Ensemble (GBM)** | Shrinkage : 0.1  n.minobsinnode : 10  n.trees : 500  interaction.depth : 9 | Shrinkage : 0.2937961  n.minobsinnode : 17  n.trees : 3618  interaction.depth : 10 | Shrinkage : 0.2937961  n.minobsinnode : 17  n.trees : 3618  interaction.depth : 10 |
| **Stacked Ensemble (RF)** | ntree : 1000  mtry : 2 | ntree : 1000  mtry : 2 | ntree : 1000  mtry : 2 |
