# Supplementary material for: Ensemble machine learning for predicting in-hospital mortality in Asian women with ST-elevation myocardial infarction (STEMI)
Source: Sci Rep. 2024 May 29;14:12378. doi: 10.1038/s41598-024-61151-x (PMC11137033; doi:10.1038/s41598-024-61151-x)
Supplement: Supplementary file 3 — Supplementary Table 3. [file 41598_2024_61151_MOESM3_ESM.docx]

**Supplementary Table 3 : Importance of selected variables for each ML algorithms**

| **Variables Importance** | **Random Forest**  (11 Variables) | **Support Vector Machines**  (12 Variables) |
| --- | --- | --- |
| 1 | **Killip class** | **Killip class** |
| 2 | **Fasting blood glucose** | **Beta blocker** |
| 3 | **Age** | **Fasting blood glucose** |
| 4 | **Systolic blood pressure** | ACE Inhibitor |
| 5 | Heart rate | **Age** |
| 6 | Triglyceride | **Systolic blood pressure** |
| 7 | HDLC | Race |
| 8 | **Beta blocker** | Lateral leads: 1, aVL, V5 to V6 |
| 9 | Fibrinolytic status | Oral hypoglycemic agent |
| 10 | **Percutaneous coronary intervention** | History of Renal Disease |
| 11 | Aspirin | Right Ventricle: ST Elevation in Lead V4R |
| 12 |  | **Percutaneous coronary intervention** |
